# Parents’ Perspectives on Participation Among Gifted and Typically Developing Children: A Pilot Study

**DOI:** 10.3390/children12081060

**Published:** 2025-08-12

**Authors:** Yael Fogel, Miri Ben Amram

**Affiliations:** 1 Department of Occupational Therapy, School of Health Sciences, Ariel University, Ariel 40700, Israel; 2 Department of Education and Teacher Training Program, Faculty of Humanities and Social Sciences, Ariel University, Ariel 40700, Israel; miriba@ariel.ac.il

**Keywords:** participation, gifted children, typically developing children, social skills, ICF-CY, Child and Adolescent Scale of Participation

## Abstract

**Background/Objectives**: Despite growing interest in giftedness, the differences in daily participation between gifted and typically developing children remain understudied and insufficiently understood. Exploring these differences may provide valuable insights into the unique needs and support required for gifted children compared to their typically developing peers. This comparative exploratory study aims to examine the differences between gifted and typically developing children’s daily participation patterns in home, school, and community environments and their parents’ perspectives and explore underlying developmental characteristics that may predict their participation. **Methods**: Parents of 215 children (8–18 years; 53% boys) in a gifted group (*n* = 136) and a matched typically developing children group (*n* = 79) completed the Five-to-Fifteen-revised questionnaire and the Child and Adolescent Scale of Participation. **Results**: We found no significant between-group differences in daily participation. However, we noted significant correlations in each group between the questionnaires’ participation domains (*r* = −0.243 to −0.460 in the gifted group, and *r* = −0.57 to −0.78 in the typically developing children group). Social and memory skills predicted 24% of the gifted children’s participation, and social and mental skills predicted 65% of the typically developing children’s participation. **Conclusions**: The results indicate similar participation patterns of gifted children and typically developing children. Social skills are a key element enabling daily participation among children in both groups.

## 1. Introduction

Giftedness in children is identified at the preschool, elementary, and secondary levels [1,2]. Although this is a multifaceted issue that should not be defined by IQ scores alone [3], significant variations in the legal definitions and identification of “giftedness” have prevented the development of international data systems, making it difficult to estimate its prevalence in the general population. Scholars generally describe *giftedness* as a complex set of psychological, behavioral, and genetic characteristics culminating in outstanding abilities in one or multiple areas [4,5]. Gifted children are capable of high levels of performance. They demonstrate potential ability or achievement in specific academic and general intellectual aptitudes, productive or creative thinking, leadership, and the performing and visual arts [6,7], and are capable of high performance compared to others of similar age, experience, and environment [7]. Due to their potential, gifted children may need more challenging and differentiated educational programs and services than general school programs usually provide [8].

### 1.1. Identification of Gifted Children in the Israeli Context

The identification of giftedness varies significantly across countries and systems, with ongoing debate regarding the definition, measurement, and implications of gifted status [3,5,6]. In Israel, giftedness is determined through a national screening and selection process conducted by the Henrietta Szold Institute on behalf of the Ministry of Education [9]. This process includes a two-stage cognitive assessment: an initial screening administered to all second- or third-grade students, followed by an advanced test of general cognitive ability for high-scoring children. Those ranking within the top 1.5% to 5.0% of their cohort are officially designated as gifted and are eligible for specialized enrichment programs [9]. These programs typically include pull-out classes or full-time gifted tracks designed to support advanced learning and socioemotional development [10,11]. This approach reflects the broader trend in gifted education to emphasize potential and contextual performance rather than fixed diagnostic thresholds [4,12].

### 1.2. Theoretical Background

Over the years, various theories and models have been developed to explain giftedness and its characteristics. According to Renzulli’s [12,13] theory, a gifted person possesses a well-defined set of three interlocking trait clusters: above-average capability, commitment to the task, and creativity. Giftedness is found in the interaction of these trait clusters. Renzulli’s model highlights that gifted and talented children possess or can develop these composite traits and potentially apply them to any area of human performance. Children developing or manifesting interactions among these clusters need a broad range of educational services that regular instructional programs do not ordinarily provide [12].

Nurture theories describe giftedness as a product of a rich learning environment, intensive training, overambitious parents, and high expectations. These theories assume that intense training is necessary for excellence in a domain—and that such training is possible only with parental support [7,14]. Moreover, parental perspectives are essential in understanding gifted children’s daily functioning. Recent findings suggested that gifted children’s ability to manage stress significantly affects their parents’ stress levels, highlighting the bidirectional nature of child–parent interactions in gifted families [15]. These insights reinforce the importance of capturing parental viewpoints when assessing real-life participation, as achieved in the present study.

### 1.3. Study Rationale

Participation is a central domain in human functioning. It occurs naturally with individuals actively involved in daily life activities (occupations) they consider to be meaningful and purposeful [16], and it is vital to the human development process and lived experience. Children improve their socioemotional, cognitive, and sensorimotor capacities when they participate in structured or less-structured activities [17]. They connect with their communities and others, acquire competencies and skills, and find meaning and purpose in life [18]. However, despite participation restrictions that can negatively affect gifted children’s current and future quality of life, studies have paid relatively little attention to their participation in life situations in home, school, and community environments [19].

Research from several fields has shown that “high-level potentialities” or “intellectually gifted” children develop sensory, language, neuropsychological, and locomotor skills at an earlier-than-expected age [20]. According to Vaivre-Douret [20], the social environment interacts with these developmental advances. In some circumstances, this interaction may increase the child’s risk for learning disabilities or socioemotional difficulties to go unaddressed, hidden behind advanced intellectual abilities [21]. These children’s unique developmental characteristics, such as high self-awareness and advanced social and emotional maturity (compared to their peers), can cause them various social and emotional problems [22].

The difficulties gifted children experience with social and emotional problems in home and educational environments may pose many problems in the future. These children may be prone to mental health conditions such as depression or anxiety [8,23]. They may become bored or disruptive in classrooms when underchallenged or may underachieve to feel accepted by their classmates or teachers [8].

Considering the available literature about gifted children’s challenges, it seems those differences would also be found in their daily participation patterns. However, no evidence in the literature has addressed daily participation patterns or their relationship with gifted children’s characteristics (compared to typically developing children). Instead, the inconclusive literature addresses other similarities and differences between gifted and typically developing children. For example, a recent study directly comparing diverse physical aspects of gifted and typically developing children showed similar self-perceived and objectively assessed fitness levels in both groups [24]. On the other hand, parents and teachers of gifted children reported more complaints about their executive functioning in everyday life [25].

Although gifted children are often described as having advanced capabilities, they may also experience socioemotional difficulties, executive functioning challenges, and mismatched environments that affect their actual engagement in everyday life. Surprisingly, little empirical evidence compares the daily participation profiles of gifted and typically developing children, particularly from the perspective of their parents, who are central informants in assessing real-life functioning. Understanding whether and how participation patterns differ between these two groups may highlight unique support needs among gifted children and help educators, clinicians, and families better tailor environments and interventions that promote optimal development and well-being.

### 1.4. Aims and Research Questions

Despite growing interest in giftedness, little is known about how gifted children participate in everyday life compared to their typically developing peers. Participation is a central domain of functioning, yet the existing literature has not adequately addressed how giftedness may affect children’s engagement in home, school, and community activities. Furthermore, few studies have explored which developmental characteristics may predict participation in this unique population. This gap underscores the need for a focused investigation that considers group differences as well as within-group variability.

Our study aims to address this gap by examining participation patterns and their predictors in gifted and typically developing children, based on parental reports. Specifically, the study aims to (1) examine whether there are differences between gifted and typically developing children in their participation in home, school, and community environments, (2) explore the relationships between developmental characteristics and daily participation within each group, and (3) identify which developmental characteristics best predict participation among gifted versus typically developing children.

As such, the research questions are as follows: (1) Are there significant differences in participation scores (total and subdomains) between gifted and typically developing children? (2) What is the nature of the correlation between developmental characteristics, as measured by the Five-To-Fifteen-Revised (5-15R) and participation, as measured by the Child and Adolescent Scale of Participation (CASP) in each group? (3) Which developmental characteristics (e.g., executive function, social skills, memory) significantly predict participation in each group? Given the study’s exploratory nature, we did not propose directional hypotheses. However, we expected that social and executive function domains would emerge as significant correlates of participation.

## 2. Materials and Methods

### 2.1. Participants

We advertised in local online groups and social media to recruit the control (typically developing children) group participants. The Israeli Ministry of Education department that handles gifted students recruited parents of gifted children to the study (gifted) group. The Ministry emailed all parents of children officially diagnosed as gifted and currently or previously participating in enrichment programs for gifted children. In Israel, standardized intelligence tests such as the Wechsler Intelligence Scale for Children (WISC) are commonly used in the process to designate a child as gifted [9]. However, individual IQ scores are not routinely disclosed due to administrative and ethical restrictions and, therefore, were not available for our study.

The inclusion criteria for all participants were parents of children aged 8 to 18 years from elementary through high school who could read and comprehend the Hebrew language (the questionnaires were written in Hebrew). An additional inclusion criterion for the gifted group was that the children had received a formal gifted diagnosis according to the Ministry of Education’s standard procedures (children aged 8 years and above, who passed the Henrietta Szold Institute’s official entrance exams). The National Institute for Research in the Behavioral Sciences administers examinations on behalf of the Ministry of Education for selection into special programs for excelling and gifted students. Administered in Grade 2 or 3 (aged 8 years and older), these examinations have two stages. In Stage 1 is an initial “filtering” examination (literacy and math) for all students. Then, the top 15% of each class take the Stage 2 examination for general cognitive ability. Students scoring in the top 1.5% to 5.0% of the Stage 2 examination are encouraged to participate in various gifted and excelling students’ programs at about 50 Israeli centers [9] (p. 89). In our study, the gifted group was not only “gifted” but also participates in specially designed enrichment activities. The exclusion criterion for all participants was a disability (motor, mental, cognitive, communication, neurodevelopmental, etc.) that might affect the comparability of the body function tests.

G*Power software 3.1 guidelines [26] determined a minimum sample size of 169 participants with a medium effect size of 0.15, power = 95, and α = 0.05. Of the 301 parents who answered the study questionnaire, 173 were parents of gifted children and 128 were parents of typically developing children. However, the 86 parents who reported disabilities in the demographic questionnaire were excluded from the study: 37 (21.4%) from the gifted and 49 (38.3%) from the control group. Thus, after applying the exclusion criteria, 215 parents answered the study questionnaire (136 gifted and 79 typically developing children’s parents). See descriptive analysis in Section 3.1.

### 2.2. Measures

After signing online consent forms, which included information on the study’s purpose, participating parents were provided links to the online questionnaires.

#### 2.2.1. Demographic Questionnaire

A short demographic questionnaire developed specifically for this study included characteristics such as the child’s gender and age and the mother’s and father’s education (academic or nonacademic). For the exclusion criterion, the questionnaire also asked about disabilities, such as a motor (e.g., motor clumsiness or cerebral palsy), physical, mental (e.g., depression, anxiety, sleep, or mental disorders like schizophrenia), and cognitive (e.g., post-stroke or head injury), communication disability with language difficulty (e.g., dyspraxia or lack of fluency), or neurodevelopmental or autistic spectrum disorder (e.g., attention deficit disorder or learning disability).

#### 2.2.2. Five-to-Fifteen Revised

The Five-to-Fifteen (FTF) Revised (5-15R) [27] is a parent report questionnaire comprising 181 items depicting developmental characteristics affecting daily functioning for 5- to 15-year-olds. Scandinavian and Finnish groups developed the revised version, extending the age to 18 years [27]. The 5-15R screens for problems or symptoms in areas such as neurodevelopmental/psychiatric or emotional, autism spectrum, attention-deficit/hyperactivity, tic, developmental coordination, oppositional defiant, conduct disorders, and learning problems [27,28].

This questionnaire covers eight domains: motor skills (17 items), executive functions (25 items), memory (11 items), perception (18 items), language (21 items), learning (29 items for children older than 8 years), social skills (27 items), and mental (emotional/behavioral) problems (33 items). Respondents score each item as 0 (*statement does not apply*), 1 (*sometimes applies or to some extent*), or 2 (*definitely applies*) [27,29]. Many studies have reported its psychometric properties, and internal consistency of the FTF domains ranged from 0.84 to 0.93. Criterion validity with the Wechsler Intelligence Scale for Children (3rd ed.) and group differential validity have been established [27]. The 5-15R was translated forward/backward into Hebrew and culturally adapted with the authors’ permission. It can be downloaded from the FTF website (https://www.5-15.org/download accessed on 11 August 2025). For this study, Cronbach’s alpha values (α = 0.97) were considered sufficient for the 5-15R total.

#### 2.2.3. Child and Adolescent Scale of Participation

The CASP [30,31] assesses participation in home, community, school, and living activities. Specifically, it measures and compares a child’s extent of participation and restrictions, as reported by a parent or caregiver, in activities compared to same-age peers. It was developed from several domains reported in the literature [32] and identified by family caregivers and rehabilitation professionals.

Recently developed based on the World Health Organization’s International Classification of Functioning, Disability, and Health: Children and Youth Version (ICF-CY) [32], a literature review, and family, child, clinician, and researcher feedback, this questionnaire assesses children’s participation [33,34,35]. The CASP broadly taps into the ICF participation domains’ classifications except for learning and applying knowledge. Specifically, it examines the domains of general tasks and demands, communication, mobility, self-care, domestic life, interpersonal interactions and relationships, major life areas, and community, social, and civic life [33,34,35].

The CASP includes 20 ordinal-scaled items in four sections (home, school, community, and living activities), each rated on a four-point scale of 4 (*age expected*), 3 (*somewhat limited*), 2 (*very limited*), and 1 (*unable*) or as “not applicable.” The summary scores (the four subsections and the total) are transformed to a 100-point scale. Specifically, scores from each applicable item are summed, divided by the maximum possible score, and multiplied by 100. Higher scores signify more age expected participation. Items 1, 6, 4, 7, 9, 2, 11, and 20 cover the ICF-CY domains [36]: 75% of all items cover participation, and 25% cover activities.

The CASP could be considered a participation-centered outcome instrument valuable for participation–intervention research [37]. It has high internal consistency (α = 0.96) [30] and good test–retest reliability (intraclass correlation coefficient α = 0.97) [37]. Construct validity has been found for the three factors [30], and discriminate validity has been found between children and youth with traumatic brain injury [38].

### 2.3. Data Analysis

We analyzed the data using IBM SPSS (ver. 26) and computed descriptive statistics for the participants’ demographic characteristics. Tests of normality (Kolmogorov–Smirnov and Shapiro–Wilk) indicated that the distribution of the dependent variables significantly deviated from normality. Therefore, between-group differences were analyzed using the non-parametric Mann–Whitney U test, with a minimum significance level of 0.05. Effect sizes were calculated using Cohen’s *d*, where 0.10 was considered small, 0.30 for medium, and 0.50 for large [36].

Partial Pearson correlations between the 5-15R and CASP scores according to Cohen [39] (small = 0.10–0.29, medium = 0.30–0.49, and large = 0.50–1.00) in each group were analyzed, controlling for father’s education, mother’s education, and child’s age. We conducted stepwise linear regression analyses to identify predictors of daily participation that significantly differed between the groups. A stepwise regression approach was chosen to explore the predictive contribution of multiple developmental characteristics while minimizing multicollinearity and overfitting risks. Given the exploratory nature of the study and the relatively large number of independent variables assessed in a modest sample size, stepwise regression allowed us to systematically identify the most significant predictors of participation in each group, providing a data-driven model that highlights key contributing factors. All FTF domains and father’s education, mother’s education, and child’s age were included in the regression analyses.

## 3. Results

### 3.1. Descriptive Analysis of Participants

Parents of 215 children (136 gifted and 79 typically developing children) answered the questionnaires. Table 1 lists the participants’ descriptive characteristics. No significant age difference emerged between groups. No correlations were found between the 5-15R or CASP domains and the participant demographic characteristics of age, gender, or father’s or mother’s education (academic and nonacademic).

### 3.2. Environment: Differences Between the Gifted and Typically Developing Children Groups

The CASP overall and scale scores showed no significant between-group differences. Table 2 summarizes the between-group differences. Given the five separate comparisons conducted on the CASP domains, we applied a Bonferroni correction to control for family-wise error. Accordingly, the significance threshold was adjusted to *p* < 0.01. Because all *p*-values exceeded this threshold, the interpretation of non-significant group differences remained unchanged.

### 3.3. Participation: Correlations in the Gifted and Typically Developing Children Groups

Table 3 presents the correlations between the 5-15R and the CASP domains. Significant medium correlations were found among the gifted group in the total participation score (*r* = −0.243 to −0.460). However, significantly strong correlations (*r* = −0.57 to −0.78) were also found in the typically developing children group’s total CASP score. Table 3 presents both groups’ correlations.

### 3.4. Predicting Daily Participation

Stepwise regression analyses were conducted for each group separately. In the gifted group, social skills predicted 21% (*R*^2^ = 0.20, *p* < 0.001), and memory predicted an additional 3% (*R*^2^ = 0.24, *p* < 0.05) of the variance in participation (Table 4). In the typically developing children group, social skills predicted 61% (*R*^2^ = 0.61, *p* < 0.001), and mental skills predicted another 4% (*R*^2^ = 0.65, *p* < 0.001) of the variance (Table 5). All demographic characteristics were added in the regression analyses, including mother’s education, father’s education, and child’s age, which were excluded during the statistical analysis.

## 4. Discussion

### 4.1. Overview of Main Findings

This study examined differences between gifted and typically developing children through parental reports of their participation in home, school, and community environments. Because our study is the first to assess daily participation in gifted children according to the ICF-CY framework, no evidence from prior studies directly supports our results. However, Carman’s [40] analysis of empirical articles (*n* = 103) that were published in 38 journals identified the most common methods for differentiating gifted and typically developing students. They found nine identification method categories, including achievement tests, academic achievements, teacher, parent, counselor, or committee recommendations, and extracurricular activities. Except for the extracurricular activities category, none directly addressed gifted children’s daily participation.

The CASP questionnaire has been found to distinguish between populations with and without cognitive challenges, such as chronic health conditions and traumatic brain injury [38]. We based our decision to use the CASP on the premise that the population of gifted children is indeed a unique population with special needs relative to their peers who are not gifted. Further, they are widely considered an at-risk population due to the various challenges they face in their educational careers [41].

We found no differences in daily participation patterns between the gifted and typically developing children in the CASP total or component scores. Possibly, the gifted children’s very high cognitive abilities do not have expression in the participation levels measured by the CASP; thus, their participation in everyday life would appear similar to their peers. These results can also be explained in three directions.

First, gifted children are indeed blessed with abilities and talents but are similar to their peers in daily functioning. They enjoy studying, they work hard to complete cognitive tasks, and they want subjects that are more difficult to enhance their intellectual capabilities [8]. The common perception that they participate better than other children may be biased. No research literature has dealt directly with gifted children’s participation, but studies have addressed their social and communicative abilities with peers and the impact on their daily functioning. For example, Pontes de França-Freitas, Del Prette, and Del Prette’s [42] empirical study compared the social capabilities of gifted children (8–12 years old) in Brazil with their typically developing peers. Their results showed that gifted children manifest elevated social competencies, including self-control, problem avoidance, and expression of positive feelings.

Papadopoulos’s [43] cross-sectional study in Greece involved 108 gifted children. Compared to same-age typically developing children samples, the gifted participants reported higher scholastic competence and better relationships with others, including peers and mothers. Good verbal abilities and language proficiency are advantages that contribute to the gifted children’s social life over time. Once they find suitable friends, these children tend to establish deep, sincere connections and often maintain lifelong relationships [44]. Many find a life partner while very young, marry young, and live stable, comfortable lives [10].

The second explanation is rooted in the global and national awareness of gifted children’s needs. In Israel, for instance, gifted children account for 1% of the student population (another 5% near the giftedness threshold are termed “excellent” and have relevant enrichment programs). The gifted children are identified based on intelligence tests and offered several enrichment programs, most commonly in the form of segregated classrooms and weekly 1-day pull-out programs in special centers [11]. More than 55 enrichment programs for gifted children operate in Israel [10]. These self-contained classrooms or out-of-school enrichment programs provide opportunities for gifted students to spend time with gifted peers, maximizing opportunities for appropriate academic challenges and building relationships [45].

Per this study’s inclusion criteria, all the gifted children in our sample attended such enrichment programs and received much social and emotional support. Our results hint at these programs’ effectiveness. Thanks to the enrichment received in the programs, the gifted children function similarly to their peers despite their unique challenges. Such children need environments with talented teachers who can ensure appropriate learning depth, pace, and stimulating activities. Highly able children must be supported in building social and emotional strength to develop as happy, independent people and fulfill their potential [9].

A third explanation might be that the parents (not the children) answered the questionnaires. The parents’ viewpoints possibly introduced some bias in the participation questionnaire responses. On the one hand, the parents may not have known what activities are expected of the age or how to compare their child’s functioning with other children’s or feared stigmatizing their gifted child as a “weirdo” [45]. They then might report normal functioning. On the other hand, the parents’ subjective answers might reflect what they would like their child’s daily level of participation to be and report results similar to that found among typically developing children. The literature documents that high expectations, especially from parents but also from teachers and classmates, hampers many gifted children (especially boys) in their development [45].

Using the CASP, we found several distinct relationships between the neurodevelopmental functions tested and participation at home, school, and community in both the gifted and typically developing children groups. The literature supports these relationships for other populations. For instance, a follow-up among children and youth with acquired brain injuries identified the Functional Skills Scale of the Pediatric Evaluation of Disability Inventory’s three domains [46] that assess children’s abilities in performing social, self-care, and mobility function activities [34]. Fogel, Josman, and Rosenblum [47] found medium-to-excellent significant correlations between participation variables (frequency and involvement) that assess expression/performance management, self-regulation, organization abilities, and activities of daily living measured by the Participation and Environment Measure for Children and Youth as well as the Child Evaluation Checklist [48].

We attempted to identify which functions would predict the children’s participation in all contexts. Unsurprisingly, the social component best predicted the participation of both groups. The difference, however, was in the degree of prediction: 61% in the typically developing children group and only 21% in the gifted group. The emotional domain contributed another 4% to the prediction among the typically developing children, whereas memory contributed another 3% to the prediction among the gifted children. The question then arises: If the social and memory function components together contributed only 24% to predicting the gifted children’s participation, what factors that may not have been examined in this study constitute the other 76%?

The explained variance in the regression model for the gifted group was relatively low (*R*^2^ = 0.24). However, this is not unexpected in behavioral and developmental research, particularly when studying complex, multifactorial outcomes such as daily participation. In contrast, the model for the typically developing group yielded a high explained variance (*R*^2^ = 0.65), suggesting a stronger relationship between developmental characteristics and participation in that group. The modest *R*^2^ value in the gifted group nonetheless highlights meaningful contributions of social and memory skills as predictors. Even when the overall explained variance is limited, identifying significant predictors can inform theory-building and guide practical interventions. These findings underscore the importance of exploring additional factors that may account for the remaining unexplained variance in participation among gifted children.

### 4.2. Implications for the Field

The ICF-CY model includes an additional part for environmental-related factors. It is certainly possible that enrichment and support services, policies, and personal attitudes of the people surrounding gifted children contribute to the children’s ability to participate. Research has indicated that ecosystem moderators [49], which include family, parents, and the school, are crucial psychosocial variables for transforming a child’s ability into extraordinary achievements and a prosperous future [50]. Especially in early childhood, parents can significantly affect their children’s development with everyday interactions, support, inspiration, and an atmosphere to promote their future outstanding outcomes [51]. Enhancing the family members’ caregiving capacity—by reducing parenting and caregiving stress—can have a powerfully influence gifted children’s developmental trajectories [19]. In line with recent work by Zanetti et al. [15], who found that gifted children’s stress regulation is closely linked to parental stress, our findings emphasize the value of considering family dynamics and caregiver perceptions when evaluating participation. Because parents serve as both observers and facilitators of their children’s engagement, their role is central in shaping daily experiences and opportunities for participation.

### 4.3. Study Limitations

This study has several limitations to consider. First, no information was available on the participants’ socioeconomic status, which may have affected the study results [40]. Second, this study refers only to the parents’ viewpoints and not to the students’ self-report. Another limitation is that the participation questionnaire, the CASP, may be less sensitive to gifted children not diagnosed with an impairment or disability. To date, the CASP has been applied for children with traumatic and other acquired brain injuries and developed based on reports from family caregivers. Each item is evaluated on a four-point scale, with 4 awarded for “age expected.” The scale leaves no possibility to respond, “above age expected.” This point could explain the “ceiling effect” obtained for this questionnaire in this study. A more detailed questionnaire, such as the Participation and Environment Measure for Children and Youth, might have provided a broader answer to contributing environmental factors. Finally, no age or gender divisions were made in light of the sample size. It is known that participation patterns change in adolescence, and that there are differences between boys and girls in this regard. Identifying age and gender differences might have shed more light on the results.

Another limitation concerns the use of stepwise regression. We chose this method to systematically identify the most relevant predictors in an exploratory context with many potential variables. Nevertheless, it is important to note that stepwise regression may produce unstable models and inflate explained variance (*R*^2^). Therefore, the resulting models should be interpreted with caution and considered as preliminary. Future studies with larger samples should adopt theory-driven hierarchical regression to validate and expand upon these findings.

An additional limitation concerns the socioeconomic differences between the gifted and typically developing children groups, as reflected in parental education levels. Given that higher socioeconomic status can influence both identification of gifted children and access to participation opportunities, this imbalance may have introduced potential bias. Although the mother’s and father’s education were included as independent variables in the regression models, they did not significantly predict participation and were excluded in the final stepwise models. Nevertheless, future research should aim to include more socioeconomically balanced samples or apply additional statistical controls to isolate the unique effects of developmental characteristics on participation.

### 4.4. Recommendations for Future Studies

The results of this study call for many follow-up studies. Given this study’s limitations, a more extensive study with the possibility of dividing the sample into age and gender groups may provide a broader picture of participation among gifted children. Follow-up studies could also examine the children’s self-perceptions of their overall functioning and participation patterns and compare them with their parents’ perceptions.

Future studies should explore additional factors that may account for the remaining unexplained variance in participation among gifted children. Identifying these factors may provide a deeper understanding of the unique participation patterns within this population.

Finally, beyond questionnaires and self-report assessments, it is essential to use follow-up research with observations and performance-based assessments to compare the participation patterns of gifted and typically developing children objectively.

### 4.5. Conclusions

Despite its limitations, this study provides evidence that gifted children participate similarly to typically developing children at home, at school, and in the community from the perspective of their parents. This study reflects the interdependent relationship between the children’s body functions and participation patterns. At the theoretical level, understanding that social skills are the main element predicting participation among gifted and typically developing children is an innovation that opens the way for further research on the subject. At the practical level, imparting social skills to all children will predict their better participation in everyday life. It is important that gifted children receive appropriate environmental support to develop and participate in everyday life similarly to what is expected of their peers. The support can be training for parents, educational staff, peers, and more. These results and conclusions allow room for further significant and influential research on the subject to advance an understanding of the population’s needs and respond appropriately.

## Figures and Tables

**Table 1:** Means, standard deviations, ranges, and frequencies for the sample’s demographic characteristics.

Variable		Group		*t*
		Gifted (*n* = 136)	Control (*n* = 79)	
		*M* (*SD*) [Range]		
**Age (years)**		**10.48 (1.97)** [8,9,10,11,12,13,14,15,16,17]	**11.09 (2.84)** [8,9,10,11,12,13,14,15,16,17,18]	**−1.88**
		**Frequency (%)**		**χ^2^**
**Gender**	**Girl**	**6**0 **(4**4**.**1**)**	41 **(**51**.**9**)**	**1.21**
	**Boy**	76 **(**55**.**9**)**	38 **(**48**.**1**)**	
**Mother’s education**	**Academic**	**126 (9**2**.**6**)**	55 **(6**9**.**6**) P**	19.90 ***
	**Nonacademic**	**1**0 **(**7**.**4**)**	24 **(3**0**.**4**)**	
**Father’s education**	**Academic**	**1**12 **(82.**4**)**	41 **(5**1**.9)**	16.06 ***
	**Nonacademic**	24 **(17.**6**)**	38 **(**48**.**1**)**	

*M* = mean; *SD* = standard deviation. *** *p* < 0.001.

**Table 2:** Environment differences between gifted and typically developing children groups in the Child and Adolescent Scale of Participation [33].

Environment	Group ^1^		*U*	*Z*	*p*
	Gifted (*n* = 136)	Typically Developing Children (*n* = 79)			
	*M* (*SD*)				
Total score	3.83 (0.23)	3.75 (0.42)	5076	−0.69	0.48
Home	3.92 (0.18)	3.83 (0.45)	4980	−1.14	0.26
Community	3.78 (0.50)	3.70 (0.56)	5027	−0.97	0.33
School	3.94 (0.15)	3.88 (0.42)	5266	−0.38	0.70
Activity	3.64 (0.56)	3.57 (0.67)	5259	−0.28	0.78

**^1^** Both groups were without disability.

**Table 3:** Participation correlations between the Child and Adolescent Scale of Participation (CASP) and the Five-to-Fifteen Revised (5-15R) domains by group.

CASP Domain ^1^	Group									
	Gifted (*n* = 136)					Typically Developing Children (*n* = 79)				
	5-15R Domain					5-15R Domain				
	Home	Community	School	Activity	Total	Home	Community	School	Activity	Total
**Motor**	−0.005	−0.12	−0.18 *	−0.25 *	−0.23 **	−0.64 ***	−0.50 ***	−0.55 ***	−0.32 **	−0.60 ***
**EF**	−0.30 ***	−0.12	−0.22 *	−0.22 **	−0.29 ***	−0.55 ***	−0.38 ***	−0.46 ***	−0.43 ***	−0.57 ***
**SPA**	−0.25 **	−0.17 *	−0.27 **	−0.17 *	−0.28 **	−0.63 ***	−0.54 ***	−0.65 ***	−0.36 **	−0.64 ***
**MEM**	−0.47 ***	−0.22 *	−0.37 ***	−0.18 *	−0.37 ***	−0.51 ***	−0.51 ***	−0.56 ***	−0.32 **	−0.57 ***
**LAN**	−0.34 ***	−0.21 *	−0.31 ***	−0.05	−0.26 **	−0.77 ***	−0.63 ***	−0.77 ***	−0.28 *	−0.73 ***
**LEARN**	−0.38 ***	−0.30 ***	−0.36 ***	−0.22 **	−0.41 ***	−0.50 ***	−0.46 ***	−0.50 ***	−0.42 ***	−0.58 ***
**SOCI**	−0.48 ***	−0.49 ***	−0.49 ***	−0.10	−0.46 ***	−0.80 ***	−0.75 ***	−0.74 ***	−0.34 **	−0.78 ***
**MENT**	−0.39 ***	−0.34 ***	−0.32 ***	−0.16	−0.38 ***	−0.77 ***	−0.72 ***	−0.77 ***	−0.36 **	−0.78 ***

^1^ EF = executive function; SPA = perception; MEM = memory; LAN = language and communication; LEARN = learning skills; SOCI = social skills; MENT = mental health problem. * *p* < 0.05; ** *p* < 0.01; *** *p* < 0.001.

**Table 4:** Predicting participation in the gifted group: Stepwise regression.

Variable ^1^	B	*SE* B	ß	B	*SE* B	ß
**SOCI**	−0.38	0.06	−0.46 ***	−0.30	0.07	−0.37 ***
**MEM**				−0.27	0.11	−0.21 *
***R*^2^ (Adjusted)**	0.21 (0.20)			0.24 (0.23)		
***F***	35.79 ***			6.05 *		

^1^ SOCI = social skills; MEM = memory. * *p* < 0.05; *** *p* < 0.001.

**Table 5:** Predicting participation in the typically developing children group: Stepwise regression.

Variable ^1^	B	*SE* B	ß	B	*SE* B	ß
**SOCI**	−1.16	0.11	−0.78 ***	−0.63	0.21	−0.42 **
**MENT**				−0.74	0.25	−0.41 **
***R*^2^ (Adjusted)**	0.61 (0.60)			0.65 (0.64)		
***F***	120.337 ***			8.45 **		

^1^ SOCI = social skills; MENT = mental health problem. ** *p* < 0.01; *** *p* < 0.001.

## Data Availability

The data presented in this study are available from the corresponding author upon request. The data are not publicly available due to ethical restrictions.

## Abbreviations

The following abbreviations are used in this manuscript:

FTF	Five-to-Fifteen
5-15R	Five-to-Fifteen Revised
CASP	Child and Adolescent Scale of Participation
ICF-CY	International Classification of Functioning, Disability, and Health: Children and Youth Version
EF	Executive functions
SPA	Perception
LAN	Language and communication
LEARN	Learning skills
SOCI	Social skills
MEM	Memory
MENT	Mental health problem
